# Supplementary material for: Distinct roles of monkey OFC-subcortical pathways in adaptive behavior
Source: Nat Commun. 2024 Aug 28;15:6487. doi: 10.1038/s41467-024-50505-8 (PMC11358305; doi:10.1038/s41467-024-50505-8)
Supplement: Supplementary file 1 — Supplementary Information [file 41467_2024_50505_MOESM1_ESM.pdf]

Supplementary Information for "Distinct roles of monkey OFC-subcortical pathways in adaptive behavior"

Kei Oyama[1,2], Kei Majima[2,3], Yuji Nagai[1], Yukiko Hori[1], Toshiyuki Hirabayashi[1], Mark A G Eldridge[4], Koki Mimura[1,5], Naohisa Miyakawa[1], Atsushi Fujimoto[1], Yuki Hori[1], Haruhiko Iwaoki[1], Ken-ichi Inoue[6], Richard C Saunders[4], Masahiko Takada[6], Noriaki Yahata[3], Makoto Higuchi[1], Barry J. Richmond[4], Takafumi Minamimoto[1]*

[1]Advanced Neuroimaging Center, National Institutes for Quantum Science and Technology, Chiba, Japan
[2]PRESTO, Japan Science and Technology Agency, Kawaguchi, Japan
[3]Institute for Quantum Life Science, National Institutes for Quantum Science and Technology, Chiba, Japan
[4]Laboratory of Neuropsychology, National Institute of Mental Health, National Institutes of Health, Bethesda, USA
[5]Research Center for Medical and Health Data Science, The Institute of Statistical Mathematics, Tachikawa, Japan
[6]Systems Neuroscience Section, Center for the Evolutionary Origins of Human Behavior, Kyoto University, Inuyama, Japan

Content of this file

1. **Figure S1**: Expression of hM4Di in the OFC$^{11/13}$ and its terminal sites.
2. **Figure S2**: Estimated inverse temperature by model fitting analysis.
3. **Figure S3**: Effects of intramuscular DCZ administration just before the reversal and without hM4Di expression on the NOVEL task performance.
4. **Figure S4**: Effect of chemogenetically silencing bilateral OFC$^{11/13}$ on the two-arm reversal learning task.

5. **Figure S5**: Effect of chemogenetically silencing bilateral $OFC^{11/13}$ on performance for the Wisconsin Card Sorting Test and the devaluation test.
6. **Figure S6**: Stability of the chemogenetic silencing of the $OFC^{11/13}$-MDm pathway during the session.
7. **Figure S7**: Effects of chemogenetically silencing the $OFC^{11/13}$-PUT pathway on NOVEL task performance and agonist injections into the non-DREADD area.
8. **Figure S8**: Overall experimental design.
9. **Figure S9**: Effects of chemogenetically silencing $OFC^{11/13}$, the $OFC^{11/13}$-rmCD pathway, and the $OFC^{11/13}$-MDm pathway on reaction time during the NOVEL task.
10. **Figure S10**: The effects of silencing $OFC^{11/13}$, the $OFC^{11/13}$-rmCD pathway, and the $OFC^{11/13}$-MDm pathway on the sensitivity to past outcomes during the FAMILIAR task.

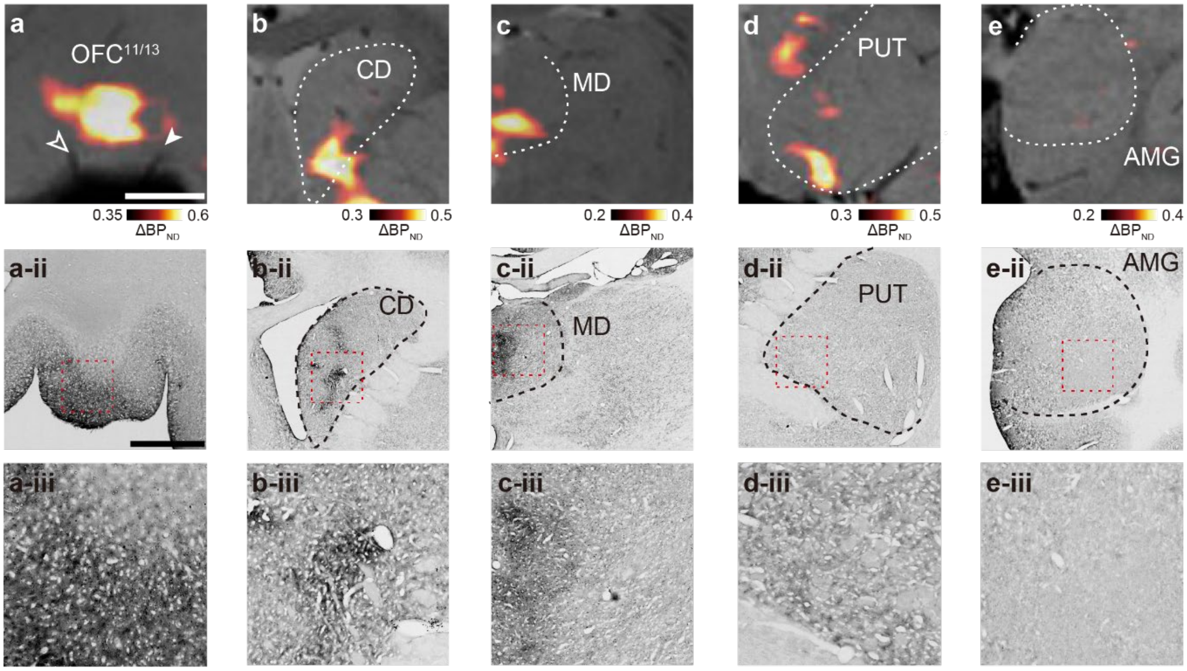

Supplementary Fig. 1 | Expression of hM4Di in the OFC$^{11/13}$ and its terminal sites.

a-e, *In vivo* visualization of hM4Di expression in the OFC$^{11/13}$ (**a**), rmCD (**b**), MDm (**c**), putamen (PUT) (**d**), and amygdala (AMG) (**e**) obtained from Mk#2. Images are coronal PET contrasts showing specific binding of [^{11}C]DCZ (contrast: after the introduction of hM4Di minus before the introduction), overlayed by MR images from Mk#2. The middle row (a-ii to e-ii) visualizes corresponding DAB-stained sections showing immunoreactivity against a reporter protein (AcGFP), and the bottom row (a-iii to e-iii) shows an enlarged view of the areas marked with red rectangles. Scale bars: 5 mm.

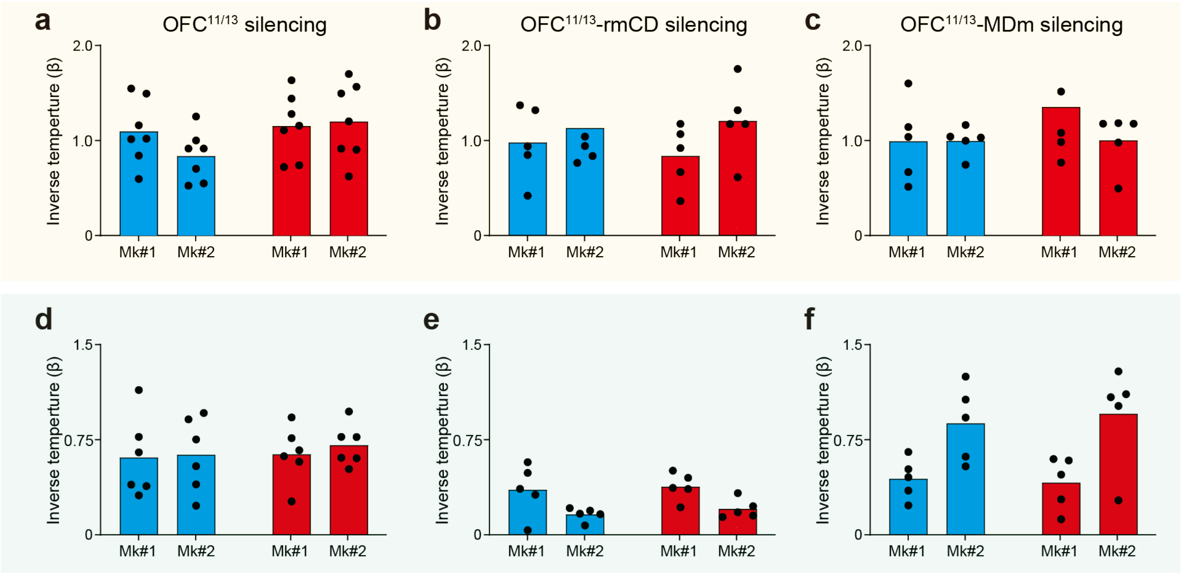

Supplementary Fig. 2 | Estimated inverse temperature by model fitting analysis.

a-c, Inverse temperatures estimated by fitting performance on the NOVEL task to the EXP model after OFC$^{11/13}$ silencing (**a**) (two-way ANOVA, treatment, $F_{(1,24)} = 2.7$, $p = 0.11$; subject, $F_{(1,24)} = 0.69$, $p = 0.41$; interaction, $F_{(1,24)} = 1.4$, $p = 0.24$), the OFC$^{11/13}$-rmCD pathway (**b**) (treatment, $F_{(1,16)} = 0.03$, $p = 0.86$; subject, $F_{(1,16)} = 1.9$, $p = 0.18$; interaction, $F_{(1,16)} = 0.33$, $p = 0.58$), or the OFC$^{11/13}$-MDm pathway (**c**) (treatment, $F_{(1,16)} = 0.94$, $p = 0.35$; subject, $F_{(1,16)} = 0.85$, $p = 0.37$; interaction, $F_{(1,16)} = 0.87$, $p = 0.37$) for control vehicle (cyan) and DCZ treatment (red) in each monkey. Only data for the post-reversal phase are shown. **d-f**, Inverse temperatures estimated by fitting to the performance on the FAMILIAR task to the INF model after OFC$^{11/13}$ silencing (**d**) (two-way ANOVA, treatment, $F_{(1,20)} = 0.21$, p = 0.65; subject, $F_{(1,20)} = 0.24$, p = 0.63; interaction, $F_{(1,20)} = 0.054$, p = 0.82), the OFC$^{11/13}$-rmCD pathway (**e**) (treatment, $F_{(1,16)} = 0.39$, $p = 0.54$; subject, $F_{(1,16)} = 11.0$, $p = 4.5 \times 10^{-3}$; interaction, $F_{(1,16)} = 0.026$, $p = 0.87$), or the OFC$^{11/13}$-MDm pathway (**f**) (treatment, $F_{(1,16)} = 0.034$, $p = 0.86$; subject, $F_{(1,16)} = 15.4$, $p = 1.2 \times 10^{-3}$; interaction, $F_{(1,16)} = 0.17$, $p = 0.68$). For the OFC$^{11/13}$ silencing (**a, d**), data were obtained from N = 7 and 6 sessions for each treatment in each monkey for the NOVEL (**a**) and FAMILIAR (**b**) tasks, respectively. For silencing of each pathway (**b, c, e, f**), data were obtained from N = 5 sessions for each treatment, each task, and each monkey.

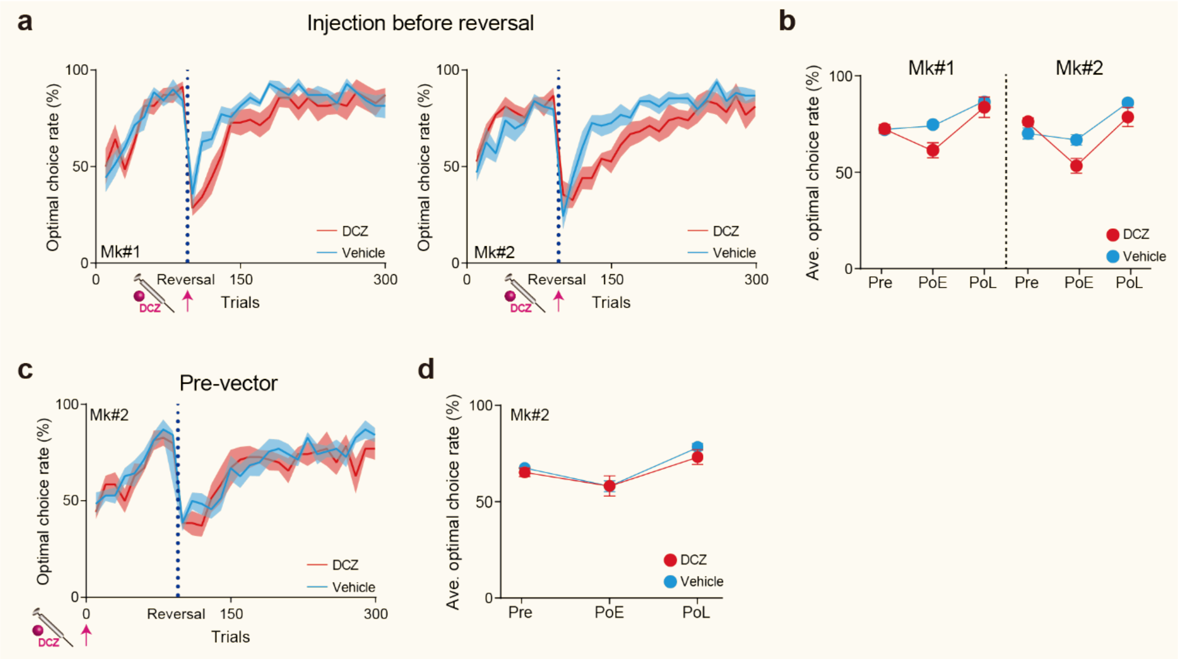

Supplementary Fig. 3 | Effects of intramuscular DCZ administration just before the reversal and without hM4Di expression on the NOVEL task performance.

a, Behavioral performance on the NOVEL task (N = 7 for each treatment in each monkey) when DCZ was administered intramuscularly just before reversal. The task was interrupted for 5 min and DCZ was administered at the start of the 5-min break. **b**, Averaged optimal choice rate for each phase of reversals for each monkey. Pre: 90 pre-reversal trials, PoE and PoL: Early and late phase of the post-reversal trials (100 and 110 trials, respectively). A three-way ANOVA (monkey × phase × treatment) revealed a significant main effect of treatment ($F_{(1,72)} = 7.9$, $p = 6.4 \times 10^{-3}$) and a significant interaction between phase and treatment ($F_{(2,72)} = 7.2$, $p = 1.3 \times 10^{-3}$). Subsequent two-way ANOVAs (monkey × treatment) for each phase revealed significant differences for treatment during the PoE ($F_{(1,24)} = 18.2$, $p = 2.7 \times 10^{-4}$) but not during the Pre ($F_{(1,24)} = 2.2$, $p = 0.15$) or the PoL ($F_{(1,24)} = 1.8$, $p = 0.19$). Error bars: s.e.m. **c**, Behavioral performance on the NOVEL task (N = 7 for each treatment, Mk#2) before the introduction of hM4Di. DCZ was administered 15 min before the beginning of the experiments as with other systemic injections. **d**, Averaged optimal choice rate for each phase of reversal. A two-way ANOVA (phase × treatment) revealed no significant differences in either treatment ($F_{(1,36)} = 0.9$, $p = 0.35$) or interaction ($F_{(2,36)} = 0.37$, $p = 0.70$). Error bars: s.e.m. In **a** and **c**, solid lines and shaded area represent the mean and s.e.m, respectively.

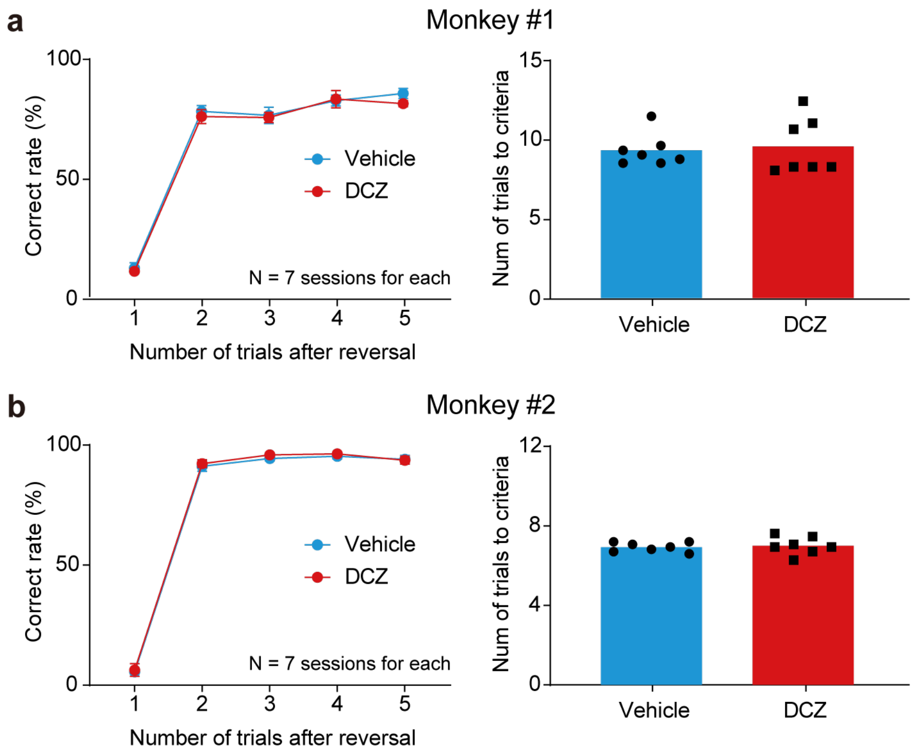

Supplementary Fig. 4 | Effect of chemogenetically silencing bilateral OFC[11/13] on the two-arm reversal learning task.

a,**b**, Correct rate as a function of the number of trials after reversal (left) and the number of trials to reach criteria (right) for Mk#1 (**a**) and Mk#2 (**b**) (N = 7 for each monkey and treatment). There was no significant difference between vehicle and DCZ injection (Two-tailed Welch's t-test, Mk#1, $t_{9.7} = 0.33$, $p = 0.75$; Mk#2, $t_{9.1} = 0.37$, $p = 0.72$). Error bars: s.e.m.

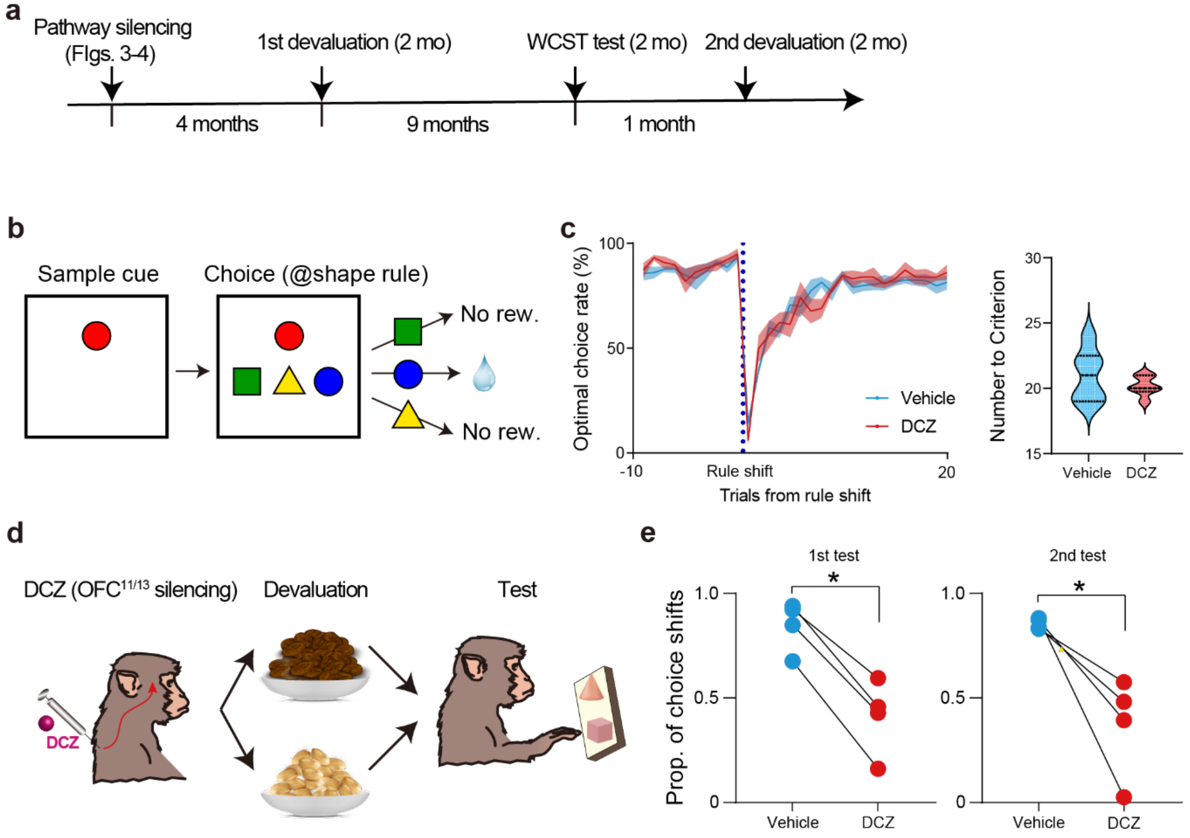

Supplementary Fig. 5 | Effect of chemogenetically silencing bilateral OFC$^{11/13}$ on performance for the Wisconsin Card Sorting Test and the devaluation test.

a, Task schedule following the pathway-selective silencing experiments shown in Fig. 3. **b**, Schema of the Wisconsin Card Sorting Test. **c**, Optimal choice rate (N = 6 for each treatment) as a function of trials for rule shift (left) and the number of trials to reach the criterion for rule shift (right). There was no significant difference in the number needed to reach criterion between vehicle and DCZ injection conditions (Two-tailed Welch's t-test, $t_{6.4} = 0.96$, $p = 0.37$). The horizontal lines in each violin plot show the quartiles of the distributions. Solid lines and shaded area represent the mean and s.e.m, respectively. **d**, Schema for the devaluation test. **e**, Performance on the devaluation test for the 1st (left) and 2nd schedule (right), respectively (N = 4 for each treatment for both schedules). There was a significant difference in performance between vehicle and DCZ injections for both schedules (Two-tailed Welch's t-test, 1st, $t_{3.1} = 4.0$, $p = 2.7 \times 10^{-2}$).

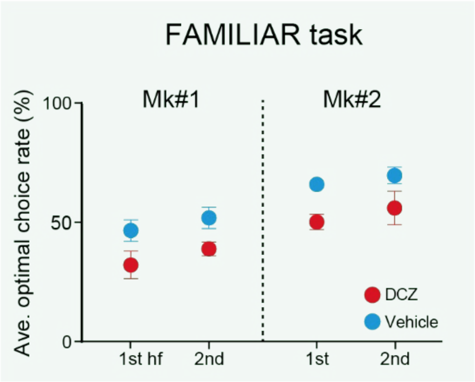

Supplementary Fig. 6 | Stability of the chemogenetic silencing of the OFC$^{11/13}$-MDm pathway during the session.

Averaged optimal choice rate in the 1st and 2nd halves of the session in the FAMILIAR task after silencing the OFC$^{11/13}$-MDm pathway. A three-way ANOVA (susbject × phase × treatment) revealed a significant main effect of treatment ($F_{(1,32)} = 20.6$, $p = 7.6 \times 10^{-5}$), but no significant interaction between treatment and phase ($F_{(1,32)} = 0.07$, $p = 0.79$). Error bars: s.e.m.

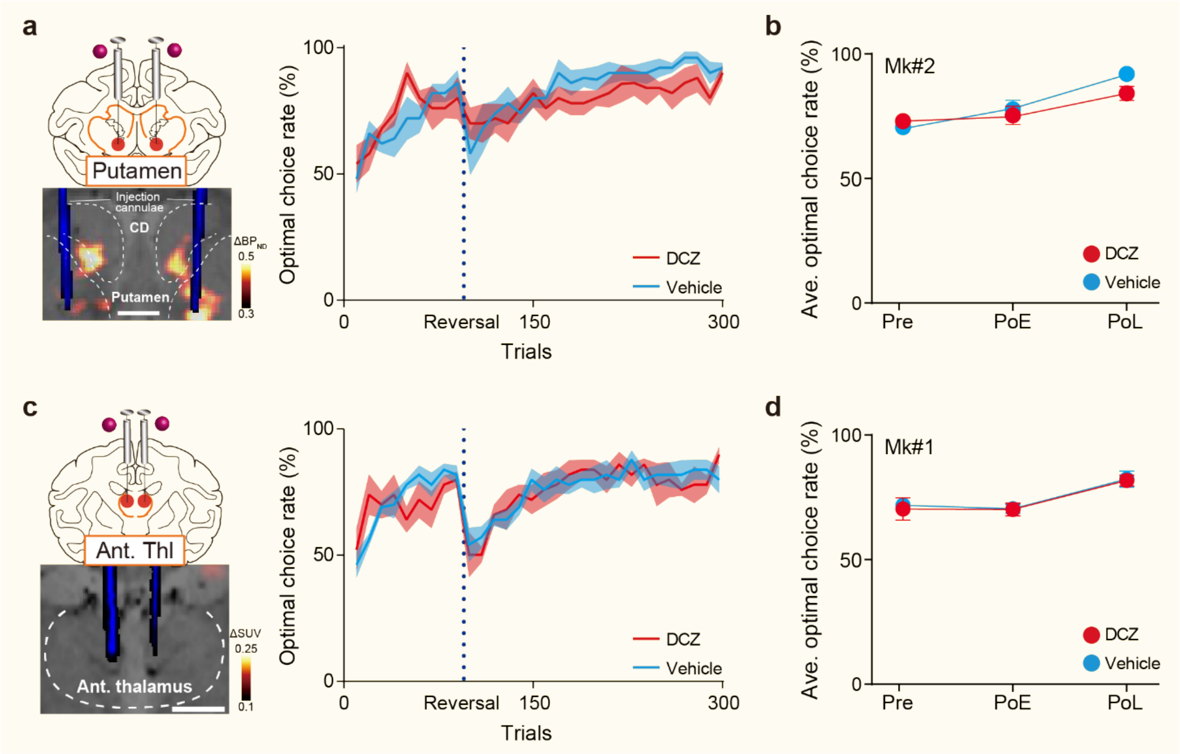

Supplementary Fig. 7 | Effects of chemogenetically silencing the $OFC^{11/13}$-PUT pathway on NOVEL task performance and agonist injections into the non-DREADD area.

a, Chemogenetically silencing the $OFC^{11/13}$-PUT pathway by local DCZ infusion into bilateral medial PUT, specifically at hM4Di-positive OFC terminals sites (top), and behavioral performance (bottom) obtained from Mk#2. N = 5 sessions for each treatment. Conventions are the same as in Fig. 2. **b**, Averaged optimal choice rate for each phase of the session. A two-way ANOVA (phase × treatment) revealed no significant main effect of treatment ($F_{(1,24)} = 1.6$, $p = 0.22$). **c**, Behavioral performance following DCZ infusion into the bilateral anterior thalamic nucleus (a region not positive for DREADDs) in the NOVEL task. N = 5 sessions for each treatment. **d**, Averaged optimal choice rate for each phase of the session. A two-way ANOVA (phase × treatment) revealed no significant main effect of treatment ($F_{(1,24)} = 0.11$, $p = 0.74$). Solid lines and shaded area in **a** and **c** represent the mean and s.e.m, respectively. Error bars: s.e.m.

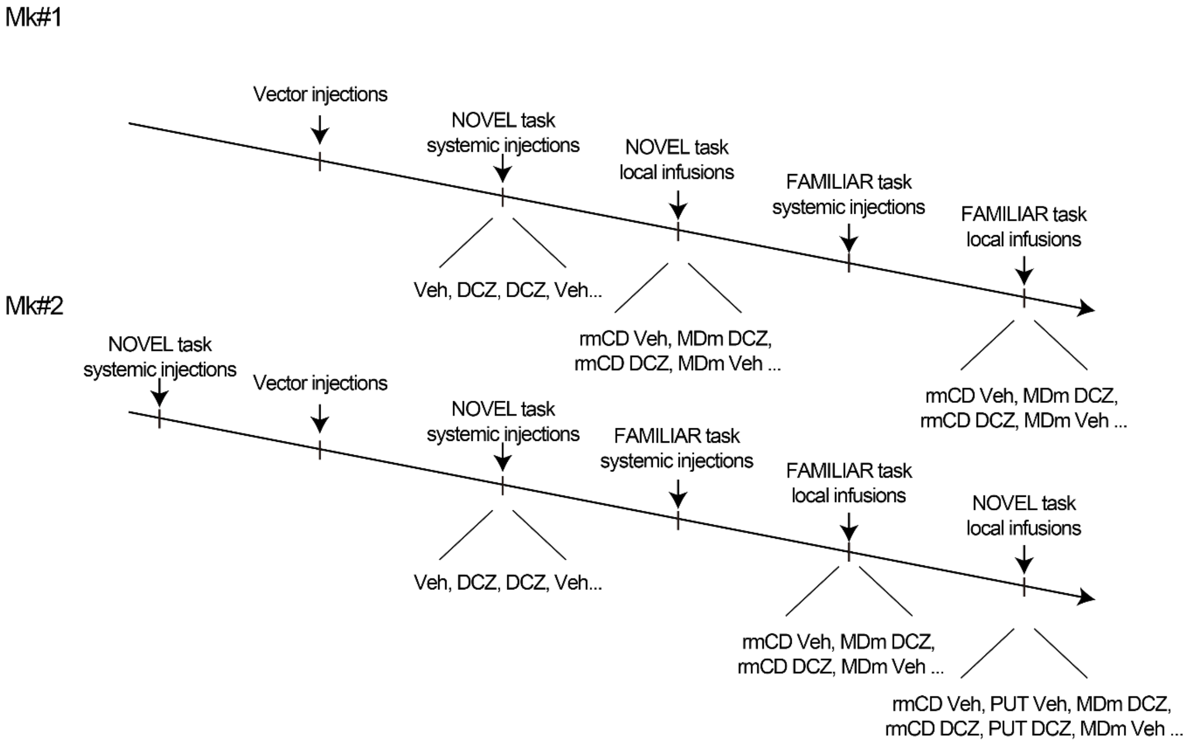

Supplementary Fig. 8 | Overall experimental design.

The sequential order of each experiment for each monkey. The order of the systemic injection type (Veh and DCZ) was pseudorandomly determined. For 4 types (MK#1, Mk#2 for the FAMILIAR task) and 6 types (Mk#2 for the NOVEL task) of local infusions, the order for the areas (rmCD, MDm, rmCD, MDm…) were constant so as to keep the same intervals of infusions into the same areas. The treatment orders were pseudorandomly determined. Note that the schedule for Mk#2 was continued to the experiments shown in Supplementary Fig. 5.

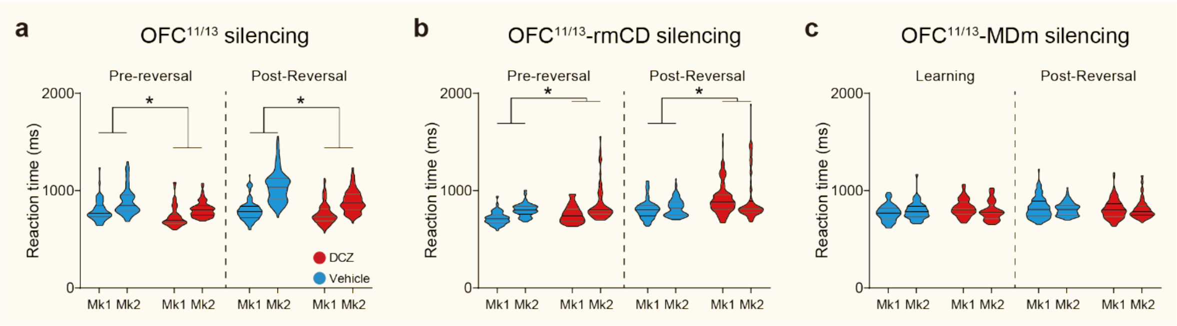

Supplementary Fig. 9 | Effects of chemogenetically silencing OFC$^{11/13}$, the OFC$^{11/13}$-rmCD pathway, and the OFC$^{11/13}$-MDm pathway on reaction time during the NOVEL task.

a-c, Reaction time in the pre-reversal phase (left) and post-reversal phase (right) after silencing OFC$^{11/13}$ (**a**), the OFC$^{11/13}$-rmCD pathway (**b**), and the OFC$^{11/13}$-MDm pathway (**c**) with DCZ treatment (red) or after control vehicle (cyan, no silencing) in each monkey. In both task phases, reaction times decreased significantly after OFC$^{11/13}$ silencing (pre-reversal phase: treatment, $F_{(1,248)} = 25.3$, $p = 9.5 \times 10^{-7}$; subject, $F_{(1,248)} = 38.0$, $p = 2.8 \times 10^{-9}$; interaction, $F_{(1,248)} = 0.62$, $p = 0.43$; post-reversal phase: treatment, $F_{(1,584)} = 74.1$, $p = 6.8 \times 10^{-17}$; subject, $F_{(1,584)} = 325.7$, $p = 3.4 \times 10^{-58}$; interaction, $F_{(1,584)} = 74.1$, $p = 7.3 \times 10^{-10}$), increased significantly after silencing the OFC$^{11/13}$-rmCD pathway (pre-reversal phase: treatment, $F_{(1,176)} = 10.5$, $p = 1.5 \times 10^{-3}$; subject, $F_{(1,176)} = 32.6$, $p = 4.7 \times 10^{-8}$; interaction, $F_{(1,176)} = 0.77$, $p = 0.38$; post-reversal phase: treatment, $F_{(1,416)} = 37.8$, $p = 1.9 \times 10^{-9}$; subject, $F_{(1,416)} = 0.34$, $p = 0.56$; interaction, $F_{(1,416)} = 0.39$, $p = 0.53$), and did not change after silencing the OFC$^{11/13}$-MDm pathway (pre-reversal phase: treatment, $F_{(1,176)} = 2.7$, $p = 0.10$; subject, $F_{(1,176)} = 0.14$, $p = 0.71$; interaction, $F_{(1,176)} = 3.7$, $p = 0.06$; post-reversal phase: treatment, $F_{(1,416)} = 0.76$, $p = 0.38$; subject, $F_{(1,416)} = 0.22$, $p = 0.64$; interaction, $F_{(1,416)} = 0.58$, p = 0.45). Although there was a significant interaction in the post-reversal phase after OFC$^{11/13}$ silencing, we found significant differences in the effects of the treatment in individual monkeys (individual Welch's t-tests; Mk#1, $t_{(291)} = 2.0$, $p = 4.5 \times 10^{-2}$; Mk#2, $t_{(261)} = 9.3$, $p = 6.2 \times 10^{-18}$), indicating that the effects were consistent across monkeys. The horizontal lines in each violin plot show the quartiles of the distributions. Asterisks: $p < 0.05$ for significant main effect of treatment. Data were averaged across 10 trials from 7 sessions for each treatment in each monkey for the OFC$^{11/13}$ silencing, and 5 sessions for each pathway silencing for Pre-reversal (90 trials) and Post-reversal (210 trials), respectively.

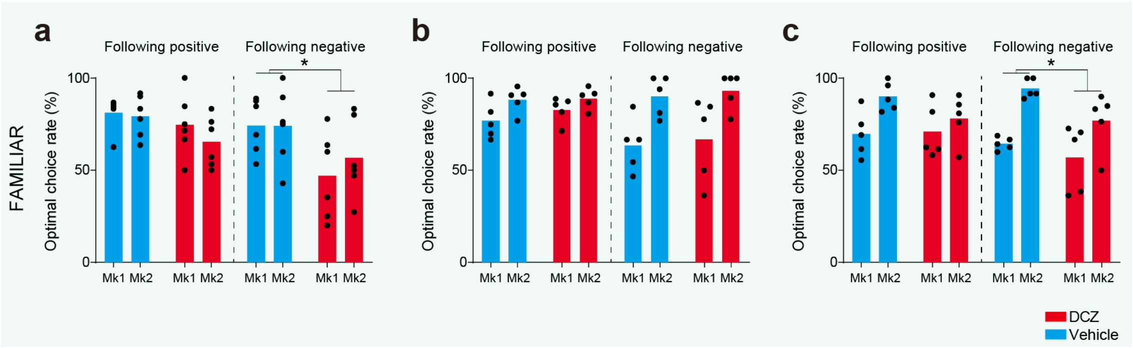

Supplementary Fig. 10 | The effects of silencing $OFC^{11/13}$, the $OFC^{11/13}$-rmCD pathway, and the $OFC^{11/13}$-MDm pathway on the sensitivity to past outcomes during the FAMILIAR task.

a-c, Averaged optimal choice rates for trials after the reversal in the FAMILIAR task following positive (left) and negative (right) outcomes after $OFC^{11/13}$ silencing (**a**) (positive: treatment, $F_{(1,20)} = 3.7$, p = 0.069; subject, $F_{(1,20)} = 1.1$, $p = 0.31$; interaction, $F_{(1,20)} = 0.47$, $p = 0.50$; negative: treatment, $F_{(1,20)} = 7.2$, $p = 1.4 \times 10^{-2}$; subject, $F_{(1,20)} = 0.33$, $p = 0.57$; interaction, $F_{(1,20)} = 0.36$, $p = 0.55$), $OFC^{11/13}$-rmCD silencing (**b**) (positive: treatment, $F_{(1,16)} = 1.0$, $p = 0.32$; subject, $F_{(1,16)} = 6.6$, $p = 2.1 \times 10^{-2}$; interaction, $F_{(1,16)} = 0.58$, $p = 0.46$; negative: treatment, $F_{(1,16)} = 0.21$, $p = 0.65$; subject, $F_{(1,16)} = 15.1$, 1.3×10^{-3}; interaction, $F_{(1,16)} = 0.0004$, $p = 0.99$), and $OFC^{11/13}$-MDm silencing (**c**) (positive: treatment, $F_{(1,16)} = 0.40$, $p = 0.54$; subject, $F_{(1,16)} = 2.6$, $p = 0.13$; interaction, $F_{(1,16)} = 0.10$, $p = 0.76$; negative: treatment, $F_{(1,16)} = 6.0$, $p = 2.6 \times 10^{-2}$; subject, $F_{(1,16)} = 18.0$, $p = 6.0 \times 10^{-4}$; interaction, $F_{(1,16)} = 0.17$, $p = 0.68$). For the $OFC^{11/13}$ silencing, data were obtained from N = 6 sessions for each treatment in each monkey. For silencing of each pathway, data were obtained from N = 5 sessions for each treatment, and each monkey.
