# Supplementary material for: Distinct roles of monkey OFC-subcortical pathways in adaptive behavior
Source: Nat Commun. 2024 Aug 28;15:6487. doi: 10.1038/s41467-024-50505-8 (PMC11358305; doi:10.1038/s41467-024-50505-8)
Supplement: Supplementary file 3 — Reporting Summary [file 41467_2024_50505_MOESM3_ESM.pdf]

Corresponding author(s): Takafumi Minamimoto

Last updated by author(s): May 28, 2024

Reporting Summary

Nature Portfolio wishes to improve the reproducibility of the work that we publish. This form provides structure for consistency and transparency in reporting. For further information on Nature Portfolio policies, see our Editorial Policies and the Editorial Policy Checklist.

Statistics

For all statistical analyses, confirm that the following items are present in the figure legend, table legend, main text, or Methods section.

n/a	Confirmed	
☐	☒	The exact sample size (*n*) for each experimental group/condition, given as a discrete number and unit of measurement
☐	☒	A statement on whether measurements were taken from distinct samples or whether the same sample was measured repeatedly
☐	☒	The statistical test(s) used AND whether they are one- or two-sided<br>*Only common tests should be described solely by name; describe more complex techniques in the Methods section.*
☐	☒	A description of all covariates tested
☒	☐	A description of any assumptions or corrections, such as tests of normality and adjustment for multiple comparisons
☐	☒	A full description of the statistical parameters including central tendency (e.g. means) or other basic estimates (e.g. regression coefficient) AND variation (e.g. standard deviation) or associated estimates of uncertainty (e.g. confidence intervals)
☐	☒	For null hypothesis testing, the test statistic (e.g. *F*, *t*, *r*) with confidence intervals, effect sizes, degrees of freedom and *P* value noted<br>*Give P values as exact values whenever suitable.*
☒	☐	For Bayesian analysis, information on the choice of priors and Markov chain Monte Carlo settings
☒	☐	For hierarchical and complex designs, identification of the appropriate level for tests and full reporting of outcomes
☒	☐	Estimates of effect sizes (e.g. Cohen's *d*, Pearson's *r*), indicating how they were calculated

Our web collection on statistics for biologists contains articles on many of the points above.

Software and code

Policy information about availability of computer code

Data collection	Data for each experiment was collected using<br>PET: microPET Focus 220 (Siemens Medical Solutions)<br>MRI: 7 tesla 400 mm/SS system (NIRS/KOBELCO/Brucker)<br>CT: Accuitomo170 (J. MORITA CO.)<br>Behavior: Inquisit (Millisecond)<br>Immunohistochemistry: Biorevo (Keyence)
Data analysis	Data were analyzed by followings<br>PET,CT,MRI: PMOD 3.7 (PMOD Technologies)<br>Behavior: Prism 9 (GraphPad), Matlab R2018b, custom-built Python code (ver. 3.9)

For manuscripts utilizing custom algorithms or software that are central to the research but not yet described in published literature, software must be made available to editors and reviewers. We strongly encourage code deposition in a community repository (e.g. GitHub). See the Nature Portfolio guidelines for submitting code & software for further information.

Data

Policy information about availability of data

All manuscripts must include a data availability statement. This statement should provide the following information, where applicable:

- Accession codes, unique identifiers, or web links for publicly available datasets
- A description of any restrictions on data availability
- For clinical datasets or third party data, please ensure that the statement adheres to our policy

The data generated in this study have been deposited in the Open Science Framework database (https://github.com/minamimoto-lab/2023-Oyama-OFC).

Research involving human participants, their data, or biological material

Policy information about studies with human participants or human data. See also policy information about sex, gender (identity/presentation), and sexual orientation and race, ethnicity and racism.

Reporting on sex and gender	No human participants were involved in this study.
Reporting on race, ethnicity, or other socially relevant groupings	No human participants were involved in this study.
Population characteristics	No human participants were involved in this study.
Recruitment	No human participants were involved in this study.
Ethics oversight	No human participants were involved in this study.

Note that full information on the approval of the study protocol must also be provided in the manuscript.

Field-specific reporting

Please select the one below that is the best fit for your research. If you are not sure, read the appropriate sections before making your selection.

☒ Life sciences ☐ Behavioural & social sciences ☐ Ecological, evolutionary & environmental sciences

For a reference copy of the document with all sections, see nature.com/documents/nr-reporting-summary-flat.pdf

Life sciences study design

All studies must disclose on these points even when the disclosure is negative.

Sample size	No statistical methods were used to pre-determine sample sizes but our sample sizes were similar to those reported in previous studies.
Data exclusions	No samples were excluded from the analysis.
Replication	Results were consistent across subjects, suggesting reproducibility of the results. In all experiments, resampling procedures also suggest reproducibility of some of the results. All attempts for replication were successful.
Randomization	In behavioral experiments, the order of drug injections (Vehcle or DCZ) was randomized.
Blinding	Blinding was not used for data collection or analysis in the animal experiments; scientific rigor is provided by replication across multiple animals and paradigms.

Reporting for specific materials, systems and methods

We require information from authors about some types of materials, experimental systems and methods used in many studies. Here, indicate whether each material, system or method listed is relevant to your study. If you are not sure if a list item applies to your research, read the appropriate section before selecting a response.

Materials & experimental systems

n/a	Involved in the study
☐	☒ Antibodies
☒	☐ Eukaryotic cell lines
☒	☐ Palaeontology and archaeology
☐	☒ Animals and other organisms
☒	☐ Clinical data
☒	☐ Dual use research of concern
☒	☐ Plants

Methods

n/a	Involved in the study
☒	☐ ChIP-seq
☒	☐ Flow cytometry
☒	☐ MRI-based neuroimaging

Antibodies

Antibodies used	GFP: rabbit anti-GFP monoclonal antibody (1:500, G10362, Thermo Fisher Scientific); biotinylated goat anti-rabbit IgG antibody (1:1,000; AB_2337963, Jackson ImmunoResearch, West Grove, PA, USA); avidin-biotin-peroxidase complex (ABC Elite, Vector Laboratories, Burlingame, CA, USA)
Validation	All commercial antibodies were validated by manufacturer, as follows:<br>G10362: https://www.thermofisher.com/antibody/product/GFP-Antibody-Recombinant-Monoclonal/G10362<br>AB_2337963: https://www.jacksonimmuno.com/catalog/products/111-065-046<br>ABC Elite: https://vectorlabs.com/products/vectastain-elite-abc-hrp-kit-standard

Animals and other research organisms

Policy information about studies involving animals; ARRIVE guidelines recommended for reporting animal research, and Sex and Gender in Research

Laboratory animals	Two male Japanese monkeys (Macaca fuscata) participated in the experiments (Mk#1: 7.2 kg; Mk#2: 6.8 kg; both aged 4 years at the beginning of experiments).
Wild animals	No wild animals were used.
Reporting on sex	Two male monkeys were used since only they were available.
Field-collected samples	No field-collected samples were used.
Ethics oversight	All experimental procedures involving the monkeys were carried out in accordance with the Guide for the Care and Use of Nonhuman primates in Neuroscience Research (The Japan Neuroscience Society; https://www.jnss.org/en/animal_primates) and were approved by the Animal Ethics Committee of the National Institutes for Quantum Science and Technology.

Note that full information on the approval of the study protocol must also be provided in the manuscript.
